# High-dose preoperative intraperitoneal erythropoietin and intravenous methylprednisolone in acute traumatic spinal cord injuries following decompression surgeries

**DOI:** 10.1515/med-2024-1105

**Published:** 2025-03-26

**Authors:** Luowen Wang, Genbing Shi, Yongjia Jin, Zongyi Mo, Zhaogan Ren, Zhanqiang Hua

**Affiliations:** Department of Orthopedics, Shanghai Electric Power Hospital, No. 937 Yan’an West Road, Changning District, Shanghai, 200050, China

**Keywords:** erythropoietin, intraperitoneal, methylprednisolone, neurologic functions, neuro restoration, neurotrauma, sphincter functions, spinal cord injury, traumatology

## Abstract

**Background:**

Methylprednisolone is preferably used in acute traumatic spinal cord injuries but its efficacy is limited. The objectives of the study were to evaluate the efficacy and safety of preoperative intraperitoneal erythropoietin plus a high dose of methylprednisolone against a high dose of methylprednisolone monotherapy in patients with traumatic spinal cord injuries.

**Methods:**

In the retrospective study, patients received preoperative intraperitoneal erythropoietin + intravenous methylprednisolone (EM cohort, *n* = 107) or methylprednisolone monotherapy (PE cohort, *n* = 140).

**Results:**

The time between decompression surgeries and injuries was 34.58 ± 6.39 h/patient (maximum: 49 h). Neurologic and sphincter functions of patients at follow-up in the EM cohort exhibited better than the preoperative neurologic and sphincter functions in the same cohort and also neurologic and sphincter functions at follow-up in the PE cohort (*p* < 0.05 for all). Higher 30-day postoperative mortality was reported in the PE cohort (43 (31%) vs 20 (19%), *p* = 0.0454) than those of the EM cohort.

**Conclusions:**

Preoperative intraperitoneal erythropoietin plus a high dose of methylprednisolone appears to have a beneficial neuroprotective effect, exhibited improved sphincter functions, and decreased mortality more than a high dose of methylprednisolone monotherapy in patients with traumatic spinal cord injuries who underwent surgeries.

## 1 Introduction

Traumatic head injuries may have acute spinal cord injuries that lead to disabilities of victims [1]. Incidence rates of acute spinal cord injuries in patients with traumatic head injuries have increased in China [2]. In addition, acute spinal cord injuries have high mortality and morbidity in patients with traumatic head injuries [3]. Therefore, proper treatment intervention(s) is required to reduce the disabilities and morbidities of victims [4].

In acute spinal cord injuries after mechanical deformities, the secondary damage is tissue necrosis [5]. Inflammation, peroxidation of lipids, and ferroptosis are important secondary damages of spinal cord injuries [6]. Methylprednisolone has preferably used in acute traumatic spinal cord injuries over the past more than 33 years [7]. However, methylprednisolone for the management of acute spinal cord injuries in traumatic head injuries is controversial since its recommendation [8]. The Chinese Association of Neurorestoratology has released the clinical therapeutic guidelines for neurorestoration after spinal cord injuries due to traumatic head injuries in 2016 [9], 2019 [10], and 2021 [11], and all have recommended high doses for methylprednisolone within 8 h of acute spinal cord injuries. The benefits of methylprednisolone monotherapy are limited for patients, and a comprehensive therapy combining multiple methods is necessary after spinal cord injuries due to traumatic head injuries [12]. However, there is a sufficient lack of evidence for routine use of erythropoietin. Low and high doses of erythropoietin promote reperfusion in neurological deterioration due to traumatic spinal cord injuries by inhibition of ferroptosis [13]. In addition, there is evidence for a superior intravenous high dose [4] or intramuscular intermediate-dose [14] of erythropoietin with a high dose of methylprednisolone for traumatic spinal cord injuries than a high dose of methylprednisolone monotherapy. The evidence regarding the efficacy of glucocorticoids in acute traumatic spinal cord injury is limited and, to many, unconvincing.

Two blinded, randomized controlled trials have studied the efficacy of glucocorticoid therapy in patients with acute traumatic spinal cord injury: the National Acute Spinal Cord Injury Study (NASCIS) II and III [15]. In 2013, based on the available evidence, the American Association of Neurological Surgeons and Congress of Neurological Surgeons stated that the use of glucocorticoids in acute spinal cord injury is not recommended [16]. Then, a Consortium for Spinal Cord Medicine similarly concluded that “no clinical evidence exists to recommend” the use of steroid therapy [17].

The objectives of the retrospective analysis were to evaluate the efficacy and safety of a preoperative single dose of intraperitoneal 5,000 units/kg erythropoietin plus a high dose of methylprednisolone against a high dose of methylprednisolone monotherapy in Chinese patients with spinal cord injuries due to traumatic head injuries who underwent decompression surgeries in a central coastal region of the People’s Republic of China.

## 2 Materials and methods

### 2.1 Inclusion criteria

Patients who had spinal cord injuries due to traumatic head injuries and received treatment(s) for the same within 8 h of traumatic injuries were included in the study. All patients underwent decompression surgical treatment (durotomy and duroplasty).

### 2.2 Exclusion criteria

Patients who had “penetrating” spinal cord injuries (due to weapons during training in the army or other official services) were excluded from the study. Patients with a known history of ischemic brain disease(s), cardiac disease(s), or kidney disease(s) were excluded from the study. The complete patient’s anamnesis was not available due to incomplete medical records.

### 2.3 Sample size calculation

The study assumed that 10% of patients would improve neurological function after surgery in follow-up, *α* = 0.05, and *β* = 0.1, and the sample size was 100.

### 2.4 Cohorts

A total of 107 patients had received a preoperative single dose of intraperitoneal 5,000 units/kg erythropoietin [9]. In addition, these patients had received 30 mg/kg intravenous bolus methylprednisolone within 15 min and a pause of 45 min. These administrations were followed by continuous infusion of 5.4 mg/kg/h methylprednisolone for 23 h [11] (EM cohort). A total of 140 patients had received 30 mg/kg intravenous bolus methylprednisolone within 15 min and a pause of 45 min before the operation. These administrations were followed by continuous infusion of 5.4 mg/kg/h methylprednisolone for 23 h [11] before the operation (PE cohort). The selection procedure for allocating patients to one of the cohorts was determined by multiple surgeons’ preferences. Patient level (clinical or other) factors and surgeons’ preferences (some surgeons consistently administer one treatment over the other) were reasons for patients received different treatments during the study period.

### 2.5 Preoperative outcome

Demographic (age, gender, and cause of injuries) and clinical parameters (trauma and neurological and sphincter functions) of patients before the operation were evaluated.

### 2.6 Surgical parameters

The time between surgeries and injuries of patients, the type of surgeries, hospital stays due to surgeries, and pre- and intra-operative and surgical parameters were evaluated. The selection of surgical procedures was typically based on radiographic assessment of spinal compression in conjunction with functions.

### 2.7 Postoperative outcomes

Postoperative outcomes (neurologic and sphincter functions) after surgeries and in the follow-up, periods were evaluated.

### 2.8 Neurologic function evaluations

The American Spinal Injury Association Impairment Scale [11] was used for the evaluation of spinal cord injuries. The grading of A to E was done as per Table 1. The experienced neurologist evaluated neurological functions with the help of radiological examinations. Clinical and radiological characters were considered for grading.

**Table 1:** The American Spinal Injury Association Impairment Scale for neurologic function evaluations

Grade	Characteristics
A	Complete injury, no sensory or motor function in S4 and S5
B	Incomplete injury, no motor functions, sensory junctions below injuries including S4 and S5; motor function not preserved >3 levels below injuries on both sides of the body.
C	Incomplete injury, motor functions are preserved below injuries with >1/2 of the key muscles below graded <3 on muscle testing (manual examinations)
D	Incomplete injury, motor functions are preserved below injuries with the least 1/2 of key muscles below graded <3 on muscle testing (manual examinations)
E	Incomplete injury, normal neurological functions

The neurologist evaluated neurological functions with the help of radiological examinations.

### 2.9 Sphincter function evaluations

An experienced urologist evaluated sphincter functions. According to neuro-urologic assessments and as per Table 2, sphincter functions were evaluated [4].

**Table 2:** Neuro-urologic assessments for sphincter function evaluations

Type of function	Characteristics
Normal function	The sphincter has electrical activity and therefore continence of urine in different pressures
Incomplete dysfunction	The sphincter has subnormal electric functions and urinary leakage
Complete dysfunction	The sphincter has no electrical activity and therefore no continence of urine

Urologist evaluated sphincter functions.

### 2.10 Postoperative complications

Postoperative complications including mortality (30-day mortality) of patients due to any reason(s) after surgeries in the follow-up period were collected and analyzed.

The time of 30 months after the completion of the operation was considered a follow-up period.

### 2.11 Statistical analysis

InStat 3.01 GraphPad Software Inc. (San Diego, CA, USA) was the choice for statistical analysis. Categorial variables are depicted as frequencies (percentages) and continuous normal with or without unequal standard deviation (SD) are depicted with mean ± SD. Not normal continuous variables are depicted as medians (Q3–Q1). The Chi-square test (*χ* ^2^-test) or Fisher exact test (in 2 × 2 table when the number of samples was <5 or the total sample size was <40) was used for statistical analysis of categorial variables. Kolmogorov and Smirnov method was used for the evaluation of the distribution of continuous variables. Mann–Whitney test was used for continuous non-normal variables between cohorts. An unpaired *t*-test was used for continuous normal variables between cohorts. All results were significant if *p* < 0.05.


**Informed consent:** Being a retrospective analysis the registration in the Chinese Clinical trial registry and consent to participate in the study were waived by the human ethics committee of the Shanghai Electric Power Hospital.
**Ethical approval:** The study was designed by the authors and it was approved (Approval number NUTCM1157 dated 25 April 2021) by the human ethics committee of the Shanghai Electric Power Hospital and the Chinese Association of Neurorestoratology. The study follows the law of China and the v2008 Declarations of Helsinki.

## 3 Results

### 3.1 Study population

**Figure 1:**
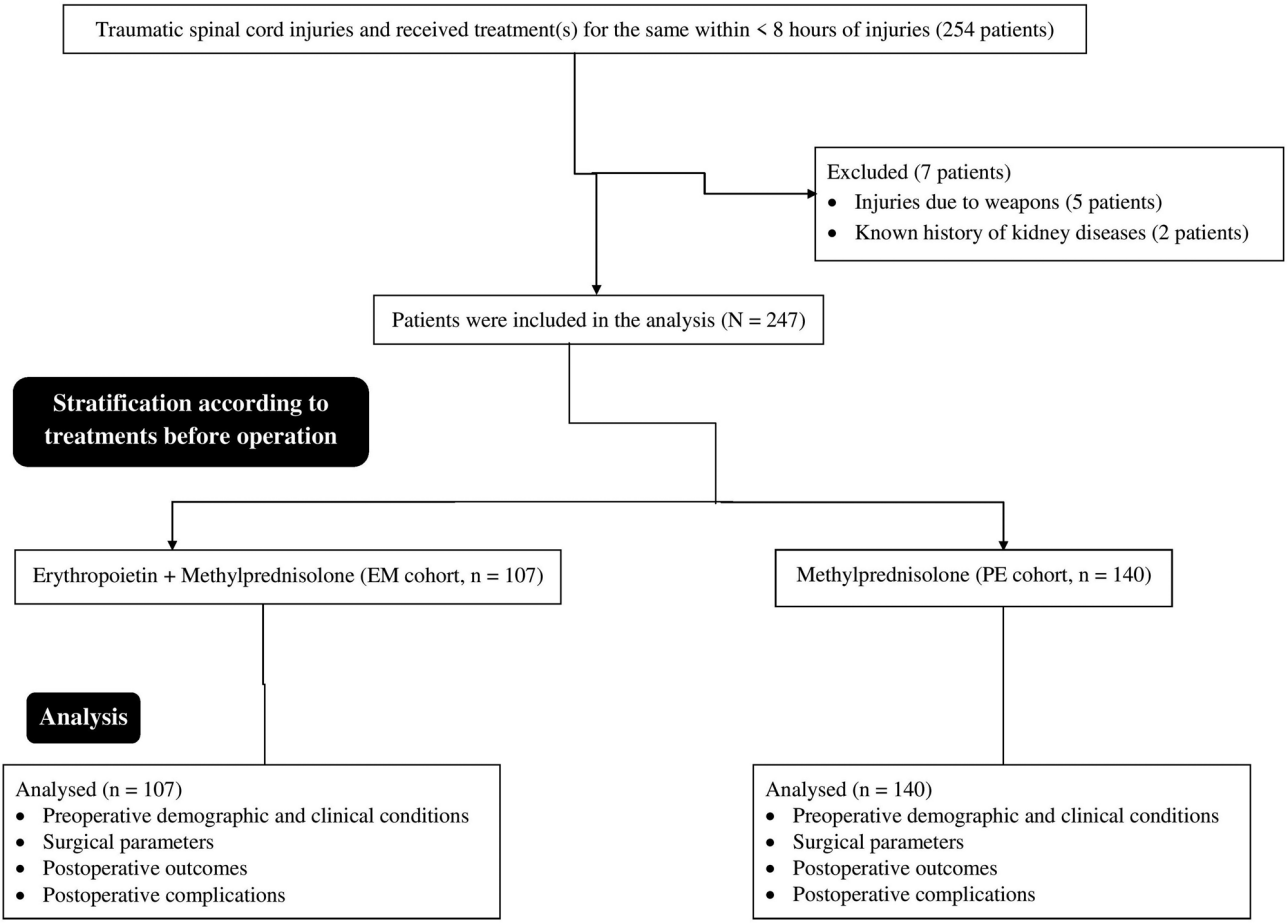
The retrospective study chart.

From May 1, 2021, to March 30, 2022, a total of 254 patients had spinal cord injuries due to traumatic injuries and received treatment(s) for the same within 8 h of traumatic injuries at the Department of Orthopedics, Shanghai Electric Power Hospital, Shanghai, China, and the referring hospitals. Among them, five patients had spinal cord injuries due to weapons, and two patients had a known history of kidney diseases. Therefore, data of these patients (seven patients) were excluded from the study. Preoperative demographic and clinical conditions, surgical parameters, postoperative outcomes, and complications in the follow-up period of 247 patients who had spinal cord injuries due to traumatic head injuries and received treatment(s) for the same within 8 h of traumatic injuries were included in the study. The retrospective study chart is presented in Figure 1.

### 3.2 Demographic and clinical parameters

Male patients were higher than female patients. Accidents were a major cause of spinal cord injuries followed by falls. None of the patients was declared dead at the time of hospital admission. There were elevations of the mean arterial pressure over 95 mmHg during the acute phase of injury in all patients. At the time of hospital admission, majorities of cases of A followed by B, C, D, and E were the American Spinal Injury Association Impairment Scale grade for neurologic functions of patients in both cohorts. At admission, majorities of patients had incomplete dysfunction followed by complete dysfunction and normal functions of the sphincter in both cohorts. Before surgeries, age, gender, cause of injuries, conditions of trauma, and neurologic and sphincter functions were comparable between cohorts (*p* > 0.05 for all, Table 3).

**Table 3:** Demographic and clinical parameters of patients before the operation at the time of hospital admission

		Cohorts					
Parameters		EM	PE
Pre-operative treatment		Erythropoietin + methylprednisolone	Methylprednisolone	Comparisons between cohorts			
Numbers of patients		107	140	*p*-value	*df*	Test value	95% CI
Age at time of injuries (years)		44 (49–38)	46 (50–37.5)	0.2753 (Mann–Whitney test)	N/A	6882.5	N/A
Gender	Male	90 (84)	123 (88)	0.4575 (Fisher’s exact test)	N/A	0.8451	0.5831–1.225
	Female	17 (16)	17 (12)				
Cause of Injuries	Accidents	80 (75)	104 (74)	0.666 (*χ* ^2^-test for independence)	2	0.666	N/A
	Fall	20 (19)	30 (22)				
	Sports	7 (6)	6 (4)				
Trauma	Head	41 (39)	62 (46)	0.6246 (*χ* ^2^-test for independence)	4	2.613	N/A
	Abdomen	15 (14)	17 (12)				
	Limbs	14 (13)	13 (9)				
	Thorax	12 (11)	21 (15)				
	More than two organs involved	25 (23)	27 (19)				
Neurologic functions	A	76 (71)	103 (73)	0.901 (*χ* ^2^-test for independence)	4	1.057	N/A
	B	8 (7)	8 (6)				
	C	8 (7)	8 (6)				
	D	8 (7)	9 (6)				
	E	7 (6)	12 (9)				
Sphincter functions	Normal function	22 (21)	43 (31)	0.1513 (*χ* ^2^-test for independence)	2	3.776	N/A
	Incomplete dysfunction	48 (45)	60 (43)				
	Complete dysfunction	37 (35)	37 (26)				

Categorial variables are depicted as frequencies (percentages) and not normal continuous variables are depicted as medians (Q3–Q1).

The neurologist evaluated neurological functions with the help of radiological examinations.

Urologist evaluated sphincter functions.

All results were significant if *p* < 0.05, *df*: degree of freedom, CI: confidence interval (using the approximation of Katz.), N/A: not applicable.

Test value (*χ* ^2^-value for *χ* ^2^-test; relive risk for Fisher’s exact test, Mann–Whitney statistics for Mann–Whitney test).

### 3.3 Surgical parameters

A total of 215 (87%) patients underwent early decompression and stabilization and 32 (13%) patients underwent durotomy and duroplasty. The type of surgeries, hospital stays due to surgeries, and pre- and intra-operative parameters were comparable between cohorts (*p* > 0.05 for all). Before the operations, if the American Spinal Injury Association Impairment Scale grade for neurological functions of patients was D or E (incomplete spinal cord injuries with or without moderately abnormal neurological functions), then they underwent durotomy and duroplasty surgeries. Before the operation, if the American Spinal Injury Association Impairment Scale grade for neurological functions of patients was A, B, or C (complete spinal cord injuries or incomplete spinal cord injuries with abnormal neurological functions), then patients underwent decompression and stabilization surgeries. In addition, patients who underwent durotomy and duroplasty surgeries or decompression and stabilization surgeries were also multiple surgeons’ preferences (radiographic assessment of spinal compression in conjunction with functions). All patients irrespective of neurological functions underwent surgeries within 3 days after injuries (time between surgeries and injuries: 34.58 ± 6.39 h/patient; maximum 49 h). The time between surgeries and injuries of patients was comparable between cohorts (34.81 ± 6.45 h/patient vs 34.41 ± 6.37 h/patient, *p* = 0.6258, unpaired *t*-test, degree of freedom: 245).

### 3.4 Postoperative outcomes

#### 3.4.1 Neurological functions

Patients receiving preoperative erythropoietin plus high dose of methylprednisolone showed improved neurological functions in follow-up after the operation as compared to those of patients before the operation and those of patients who received a high dose of methylprednisolone monotherapy in follow-up after the operation. In follow-up after the operation, majorities of patients had A followed by E, D, C, and B in the American Spinal Injury Association Impairment Scale grade for neurological functions in both cohorts. In the PE cohort, the neurologic functions of patients improved in follow-up after the operation as compared to those before the operation, but differences were not statistically significant. The details of the neurological functions of patients are reported in Table 4.

**Table 4:** Neurological functions of patients before operation and in follow-up after the operation

The American Spinal Injury Association Impairment Scale grade	Cohorts												
	EM					PE					Comparisons between cohorts at EL		
Pre-operative treatment	Erythropoietin + methylprednisolone					Methylprednisolone					Cohorts		
Level	BL	EL	^#^ *p*-value	*df*	*χ* ^2^-value	BL	EL	^#^ *p*-value	*df*	*χ* ^2^-value	*p*-value	*df*	*χ* ^2^-value
Numbers of patients	107	107				140	140						
A	76 (72)	47 (44)	0.0021	4	16.78	103 (73)	94 (67)	0.8347	4	1.454	0.0093	4	13.437
B	8 (7)	12 (11)				8 (6)	9 (6)						
C	8 (7)	14 (13)				8 (6)	10 (7)						
D	8 (7)	16 (15)				9 (6)	12 (9)						
E	7 (7)	18 (17)				12 (9)	15 (11)						

The neurologist evaluated neurological functions with the help of radiological examinations.

Variables are depicted as frequencies (percentages).

*χ* ^2^-test for independence was used for statistical analysis purposes.

*df*: degree of freedom.

^#^Between BL and EL.

BL: Before the operation, EL: in follow-up after the operation.

All results were significant if *p* < 0.05.

#### 3.4.2 Sphincter function

Patients receiving preoperative erythropoietin plus methylprednisolone showed improved sphincter function in follow-up after the operation as compared to those of patients before the operation and those of patients who received methylprednisolone monotherapy in follow-up after the operation. In follow-up after the operation, majorities of patients had incomplete dysfunctions followed by normal functions and complete dysfunction of the sphincter in both cohorts. Preoperative erythropoietin plus methylprednisolone administration improved sphincter functions from complete dysfunction to incomplete dysfunction. In addition, preoperative erythropoietin plus methylprednisolone administration improved sphincter functions from incomplete dysfunction to normal functions. However, preoperative methylprednisolone monotherapy administration did not improve sphincter functions from incomplete dysfunction to normal functions nor from complete dysfunction to incomplete dysfunction. The details of the sphincter functions of patients are reported in Table 5.

**Table 5:** Sphincter functions of patients before operation and in follow-up after the operation

Type of sphincter functions	Cohorts										Comparisons between cohorts at EL		
	EM					PE							
Pre-operative treatment	Erythropoietin + methylprednisolone					Methylprednisolone							
Level	BL	EL	^#^ *p*-value	*df*	*χ* ^2^-value	BL	EL	^#^ *p*-value	*df*	*χ* ^2^-value	*p*-value	*df*	*χ* ^2^-value
Numbers of patients	107	107				140	140						
Normal function	22 (21)	42 (39)	<0.0001	2	22.236	43 (31)	43 (31)	0.9891	2	0.022	0.0045	2	10.802
Incomplete dysfunction	48 (45)	55 (51)				60 (43)	61 (44)						
Complete dysfunction	37 (34)	10 (10)				37 (26)	36 (25)						

Urologist evaluated sphincter functions.

Variables are depicted as frequencies (percentages).

*χ* ^2^-test for independence was used for statistical analysis purposes.

^#^Between BL and EL.

BL: Before the operation, EL: follow-up after the operation, *df*: degree of freedom.

All results were significant if *p* < 0.05.

#### 3.4.3 Postoperative complications

All patients experience nausea, vomiting, dizziness, and urinary catheterization. All patients required fentanyl injection for pain management. A total of 28 (26%) patients from the EM cohort and 45 (32%) patients from the PE cohort were required for admission to the intensive care unit in follow-up after the operation. A total of 20 (19%) patients from the EM cohort and 43 (31%) patients from the PE cohort died after the operation during the follow-up period (30-day mortality). Higher postoperative mortality was reported in the PE cohort than those in the EM cohort. A total of 4 (4%) of patients from the EM cohort suffered from hypostatic pneumonia with 30 days of follow-up. A total of 2 (2%) patients from the EM cohort and 1 (1%) patient from the PE cohort experienced gastrointestinal bleeding. A total of 1 (1%) patient from the EM cohort experienced infectious complications. The details of postoperative complications in the follow-up period of 30 months after the completion of the operation are presented in Table 6.

**Table 6:** Postoperative complications in the follow-up period of 30 months after the completion of the operation

Events	Total	Cohorts		Comparisons between cohorts			
		EM	PE				
Pre-operative treatment	–	Erythropoietin + methylprednisolone	Methylprednisolone				
Numbers of patients	247	107	140	*p*-value	*df*	Test value	95% CI
Admission to intensive care unit	73 (30)	28 (26)	45 (32)	0.3974 (*χ* ^2^-test with Yates correction)	1	0.7727	0.6053–1.179
Mortality (30-day mortality)	63 (26)	20 (19)	43 (31)	0.0454 (*χ* ^2^-test with Yates correction)	1	4.003	0.4532–0.9946
Hypostatic pneumonia (with 30-day of follow-up)	4 (2)	4 (4)	0 (0)	0.0341 (Fisher’s exact test)	N/A	2.359	2.037–2.732
Gastrointestinal bleeding	3 (1)	2 (2)	1 (1)	0.5804 (Fisher’s exact test)	N/A	1.549	0.6869–3.494
Infectious complications	1 (1)	1 (1)	0 (0)	0.4332 (Fisher’s exact test)	N/A	2.321	2.010–2.679
Pressure score	35 (14)	15 (14)	20 (14)	0.9999 (Fisher’s exact test)	N/A	0.9876	0.6538–1.492

Variables are depicted as frequencies (percentages).

Patients had one or more complications.

All results were significant if *p* < 0.05.

*df*: degree of freedom, CI: 95% confidence interval (using the approximation of Katz.), N/A: not applicable.

Test value (*χ* ^2^-value for *χ* ^2^-test; relative risk for Fisher’s exact test).

## 4 Discussion

Neurological functions at follow-up in the EM cohort were better than the preoperative scores in the same cohort and also scores at follow-up in the PE cohort. The results of the neurological functions of the current study are not consistent with those of pilot trials [4,18] and a phase III trial [19] but consistent with those of a retrospective analysis [14], observation study on ischemia–reperfusion injury [20], and a case report [21]. The small sample size and different demographic, clinical, and operative parameters of patients of pilot trials [4,18] and phase III trials [19] are responsible for contradictory results. Erythropoietin inhibits the apoptosis of neurons and improves mesenchymal stem cells at the injured site [14]. These decrease the impairment of neurological functions [22]. From the current study, it would be predicted that a preoperative intraperitoneal high dose of erythropoietin plus a high dose of methylprednisolone appears to have a beneficial neuroprotective effect than a preoperative high dose of methylprednisolone monotherapy in patients with traumatic spinal cord injuries who underwent decompression surgeries within 49 h. Multicentric randomized controlled trials are needed to confirm effects.

In the current study, a preoperative intraperitoneal high single dose of erythropoietin was administered. However, in pilot trials [4,18], a phase III trial [19], and retrospective analysis [14], patients had received intravenous multiple doses of erythropoietin. Regarding the dosage, the majority of studies typically employ doses around 500 units/kg, administered multiple times, with evidence suggesting that multiple treatments yield better outcomes. Long-term administration of intravenous erythropoietin could cause thromboembolism and stroke [4,21]. A single preoperative intraperitoneal administration of a high dose of erythropoietin is safe and effective for neuroprotective actions in patients with traumatic spinal cord injuries.

Sphincter functions at follow-up in the EM cohort were better than the preoperative sphincter functions in the same cohort and also sphincter functions at follow-up in the PE cohort. In addition, in follow-up after the operation, fewer 30-day postoperative mortality was reported in the EM cohort than those in the PE cohort. The results of the sphincter functions and 30-day postoperative mortality of the current study are not consistent with those of a pilot trial [4]. The small sample size and different demographical, clinical, and operative parameters of patients of a pilot trial [4] are responsible for contradictory results. The observed higher mortality rates in the methylprednisolone monotherapy cohort may stem from severe head injuries rather than the treatment’s efficacy. Preoperative intraperitoneal high dose of erythropoietin plus a high dose of methylprednisolone appears to have more positive effects on sphincter functions and decreases postoperative mortality than a preoperative high dose of methylprednisolone monotherapy in patients with traumatic spinal cord injuries.

All patients irrespective of preoperative neurological functions underwent surgeries within 34.58 ± 6.39 h/patient (maximum: 49 h) after injuries. Inabilities of the transport system, lack of facilities for diagnosis, and absence of experts in the parent hospitals were reasons for the late performance of surgeries (within 49 h after injuries). However, the surgical window for decompression and internal fixation is less than 1 day [23]. Early decompression (<8 h) after traumatic cervical spinal cord injury improves functional outcome [24]. However, decompression surgical intervention(s) within 3 days would provide benefits from adverse neurological and postoperative effects [25]. Decompression surgery should perform as early as possible after traumatic spinal cord injuries for better post-surgical benefits.

Neurological and sphincter functions at follow-up in the PE cohort were better than the preoperative neurological and sphincter functions in the same cohort, but differences were not statistically significant. The results of postoperative outcomes of patients in the PE cohort in the current study were consistent with those of a systematic review [7]. However, in pilot trials [4,18], phase III trial [19], and a retrospective analysis [14], preoperative high-dose methylprednisolone monotherapy improved the neurological and sphincter functions of patients in follow-up after the operation as compared to those before the operation with statistical differences. The small sample size and different demographical, clinical, and operative parameters of patients of pilot trials [4,18] and a phase III trial [19] are responsible for contradictory results. Preoperative high-dose methylprednisolone monotherapy appears to have a small positive benefit on postoperative outcomes of patients with traumatic spinal cord injuries after decompressive surgeries.

All patients have small or larger somewhat postoperative treatment-emergent complications. In addition, the EM cohort reported hypostatic pneumonia. High-dose methylprednisolone therapy is responsible for experiences of postoperative complications in patients. Moreover, high-dose methylprednisolone therapy combined with a high dose of methylprednisolone is burdened with the likelihood of hypostatic pneumonia.

Studying with a large number of patients is difficult in traumatic spinal cord injury studies and conclusions are supported by the data. In addition, the study with rigorous methodology and robust data could contribute valuable information to neuro-restoration after traumatic spinal cord injuries. However, there are limitations of the study, for example, retrospective analysis and lack of trial. The study evaluated neurological functions, sphincter functions, and postoperative mortality only. The other pre- and postoperative parameters were not evaluated. There are no data presented on motor or sensory score changes. There are no data presented for grade conversion based on presenting grade – only how many of the American Spinal Injury Association Impairment Scales were present initially and at follow-up. There is a lack of standardized surgical treatment. It is mentioned that some patients underwent duroplasty, but it is unclear why this was the case and when this procedure was chosen over others. Standardization of treatment protocols is important for consistency in results and reliable comparisons. The neurologic assessment using the American Spinal Injury Association Impairment Scale is a crude measure and may not capture the nuanced improvements or deteriorations in patient outcomes. More comprehensive and sensitive neurological assessments are recommended for such studies. Most importantly, the study reports a high mortality rate in the methylprednisolone monotherapy group. However, the reasons for these deaths are not adequately explained. Understanding these reasons is vital for assessing the safety and risks associated with the treatments. The mortality data presented are remarkably high in both cohorts.

## 5 Conclusions

A preoperative intraperitoneal high dose of erythropoietin plus a high dose of methylprednisolone appears to have a beneficial neuroprotective effect, improves sphincter functions, and decreases postoperative mortality than a high dose of methylprednisolone monotherapy in patients with traumatic spinal cord injuries who underwent decompression surgeries within 49 h after traumatic injuries. Decompression surgery should be performed by surgeons as early as possible after traumatic spinal cord injuries for better post-surgical benefits. Preoperative high-dose methylprednisolone monotherapy would have a small positive benefit on postoperative outcomes and postoperative complications of patients with traumatic spinal cord injuries. The current study offers significant contributions to the understanding of traumatic spinal cord injury treatment and provides a robust base for further investigations in this field. Long-term outcomes, different dosing regimens, or other therapeutic combinations would be future research work.

## Abbreviations

NASCIS: The National Acute Spinal Cord Injury Study
EM cohort: Patients received preoperative intraperitoneal 5,000 units/kg erythropoietin + 30 mg/kg intravenous bolus methylprednisolone within 15 min and a pause of 45 min + continuous infusion of 5.4 mg/kg/h methylprednisolone for 23 h
PE cohort: Patients received 30 mg/kg intravenous bolus methylprednisolone within 15 min and a pause of 45 min + continuous infusion of 5.4 mg/kg/h methylprednisolone for 23 h before the operation
SD: Standard deviation
*χ* ^2^-test: Chi-square test
